# Supplementary material for: An In Silico Methodology That Facilitates Decision Making in the Engineering of Nanoscale Protein Materials
Source: Int J Mol Sci. 2022 Apr 29;23(9):4958. doi: 10.3390/ijms23094958 (PMC9099527; doi:10.3390/ijms23094958)
Supplement: Supplementary file 1 [file ijms-23-04958-s001.zip › ijms-1686926-supplementary.pdf]

A

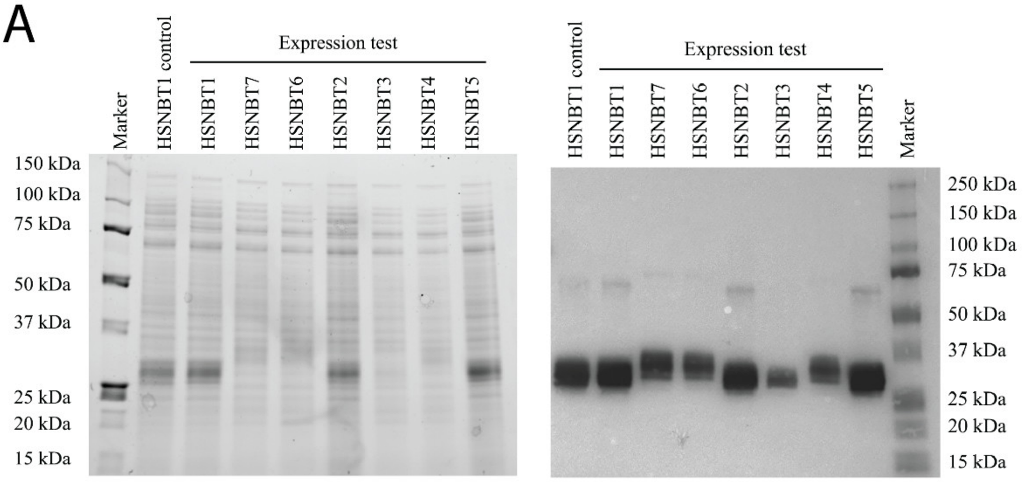

B

Mutant	Culture growth (OD)	Protein yield (mg/L)
T22-HSNBT1-H6	4.10-5.32	14-24
T22-HSNBT2-H6	3.52	9
T22-HSNBT3-H6	4.07	Not purified
T22-HSNBT4-H6	4.13	Not purified
T22-HSNBT5-H6	4.52	15
T22-HSNBT6-H6	3.28	26
T22-HSNBT7-H6	2.60-3.78	12-24
T22-HSNBT8-H6	3.17-3.54	20-28

C

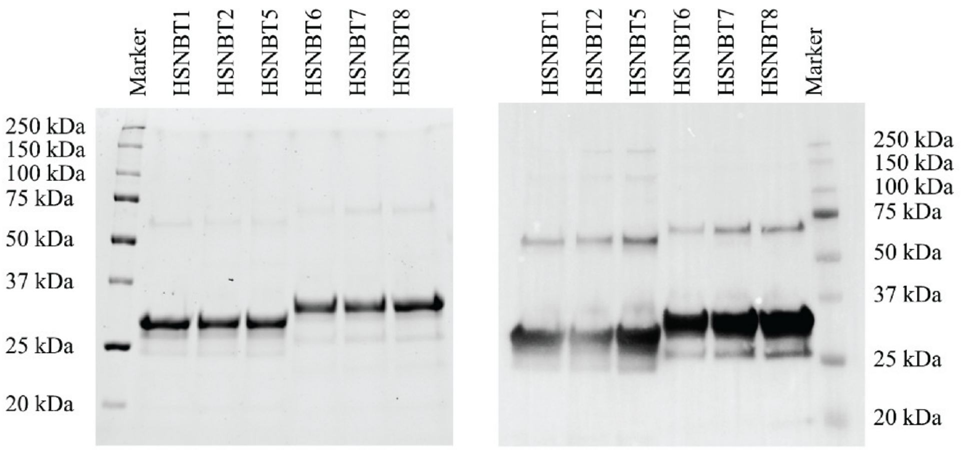

Figure S1. A. SDS-PAGE (left) and western blot (right) of the soluble fraction of cell lysates of the expression test. B. Culture growth (OD) and protein production yield of all the protein mutants derived from T22-HSNBT1-H6. C. SDS-PAGE (left) and western blot (right) of the purified proteins after production at Erlenmeyer-scale.

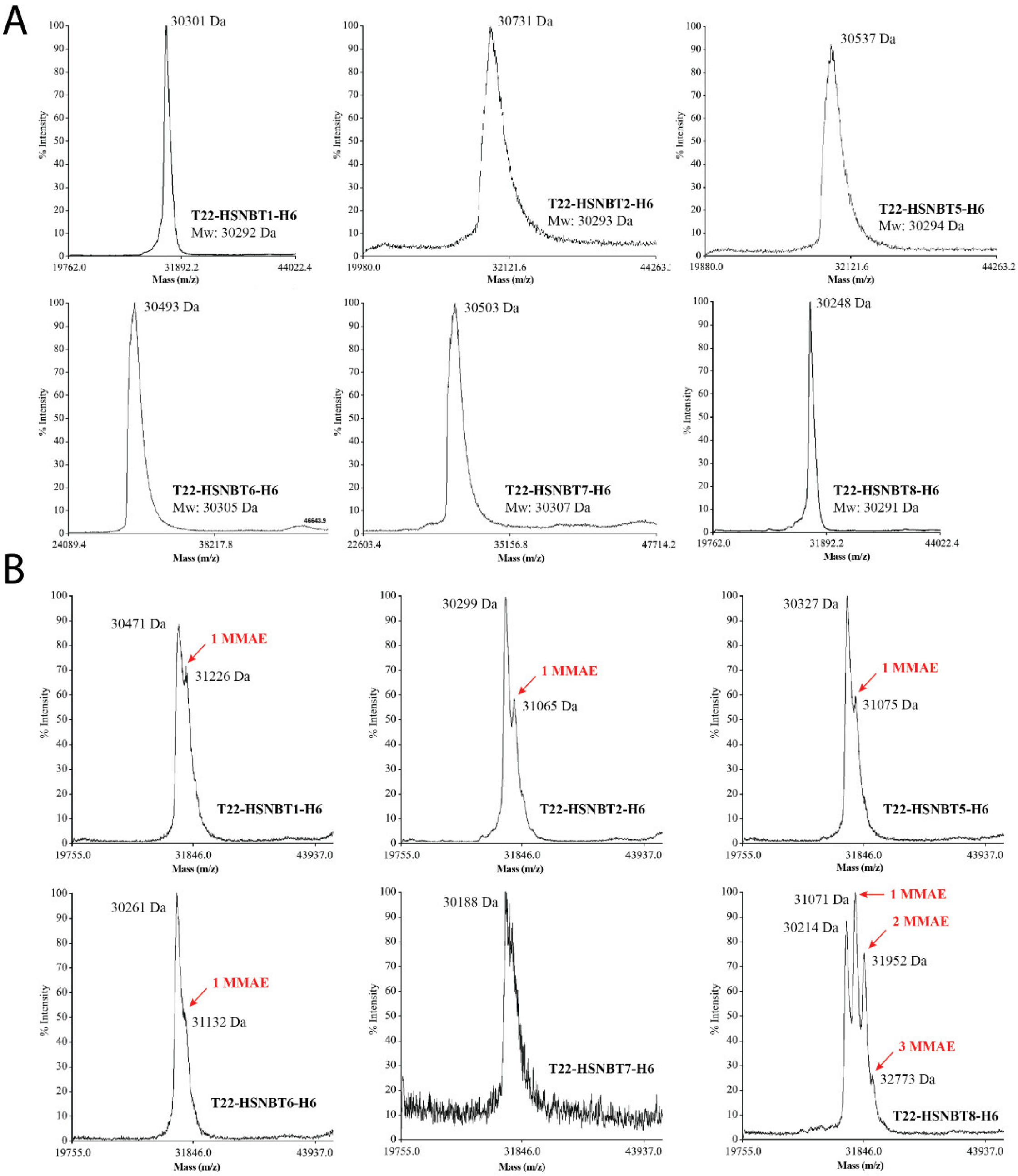

Figure S2. A. MALDI-TOF mass spectrometry spectrum of the purified candidate proteins after the expression test. The theoretical size of all proteins is approximately 30.3 kDa. B. MALDI-TOF mass spectrometry spectrum of the candidate proteins after conjugation with MMAE (soluble fraction). Red arrows indicate each peak with additional ~1 kDa over the expected protein weight, belonging to a conjugated protein with an extra MMAE (911 Da) attached.
